# Liensinine- and Neferine-Induced Cardiotoxicity in Primary Neonatal Rat Cardiomyocytes and Human-Induced Pluripotent Stem Cell-Derived Cardiomyocytes

**DOI:** 10.3390/ijms17020186

**Published:** 2016-01-29

**Authors:** Yangyang Yu, Shennan Sun, Shifeng Wang, Qiao Zhang, Ming Li, Feng Lan, Shiyou Li, Chunsheng Liu

**Affiliations:** 1 School of Chinese Materia Medica, Beijing University of Chinese Medicine, No. 6 Wangjing Zhong Huan South Road, Chaoyang District, Beijing 100102, China; louisyang@bucm.edu.cn (Y.Y.); wangshifeng@bucm.edu.cn (S.W.); zhangqiao@bucm.edu.cn (Q.Z.); 2 Beijing Institute of Genomics, Chinese Academy of Sciences, No. 1 Beichen West Road, Chaoyang District, Beijing 100101, China; pkssn12@gmail.com; 3 First Affiliated Hospital, Heilongjiang University of Chinese Medicine, Heping Road, Xiangfang District, Harbin 150040, China; lizixuanbaba@gmail.com; 4 Beijing Institute of Heart Lung and Blood Vessel Disease, 2 Anzhen Road, Chaoyang District, Beijing 100029, China; fenglan@ccmu.edu.cn

**Keywords:** liensinine, neferine, cardiotoxicity, hiPS-CM, real-time analysis

## Abstract

Due to drug-induced potential congestive heart failure and irreversible dilated cardiomyopathies, preclinical evaluation of cardiac dysfunction is important to assess the safety of traditional or novel treatments. The embryos of *Nelumbo nucifera* Gaertner seeds are a homology of traditional Chinese medicine and food. In this study, we applied the real time cellular analysis (RTCA) Cardio system, which can real-time monitor the contractility of cardiomyocytes (CMs), to evaluate drug safety in rat neonatal CMs and human induced pluripotent stem cell-derived cardiomyocytes (hiPS-CMs). This study showed detailed biomechanical CM contractility *in vitro*, and provided insights into the cardiac dysfunctions associated with liensinine and neferine treatment. These effects exhibited dose and time-dependent recovery. Neferine showed stronger blocking effect in rat neonatal CMs than liensinine. In addition, the effects of liensinine and neferine were further evaluated on hiPS-CMs. Our study also indicated that both liensinine and neferine can cause disruption of calcium homeostasis. For the first time, we demonstrated the potential cardiac side effects of liensinine or neferine. While the same inhibition was observed on hiPS-CMs, more importantly, this study introduced an efficient and effective approach to evaluate the cardiotoxicity of the existing and novel drug candidates.

## 1. Introduction

Neferine and liensinine are isoquinoline alkaloids isolated from the green seed embryos *Nelumbo Nucifera* Gaertn*,* which display multiple bioactivities including antidepressant-like action, relaxation on vascular smooth muscle and antiarrhythmic action [1,2,3,4,5]. Though the two alkaloids share similar chemical structure, the structure-activity relationships between liensinine and neferine has remained elusive. Moreover, there are reports suggesting that liensinine and neferine reverses resistance to carboplatin in Tca8113/CBP cells [6], but little is known about the cardiac contractility in liensinine and neferine.

Cultured primary cardiomyocytes and human induced pluripotent stem cell derived cardiomyocytes (hiPS-CMs) provided useful models to understand the cardiovascular function and cardiovascular diseases *in vitro* on real-time cell analysis system (RTCA), which provide a homogeneous population of relatively pure single cells [7]. This platform has been used for investigation of contraction, hypoxia and cardiotoxicity [8,9,10]. The RTCA cardio instrument provided a real-time, label-free, and non-invasive analysis of cardiomyocyte functions [11,12], which has been used in cardiovascular toxicity screening, drug-induced cardiac contractility evaluation and estimating the risk of drug-induced proarrhythmia [13,14,15].

Primary neonatal rat CMs reduce the number of animal experiments and improve the confidence in drug research. However, a sensitive tool with accurate recording capacity is required, many of the techniques only defined endpoint measurements. hiPS-CMs provided an opportunity to the routine preclinical drug evaluation, which display expected contractile characteristics of native cardiomyocytes, relevant cardiac disease phenotypes and genetically relevant background [16,17,18]. Meanwhile, the hiPS-CMs shows stable electrophysiological and contractile characteristics as expected [16,19], which enables wide range of applications, including toxicity testing, drug discovery and cardiac disease research.

Ca^2+^ is the link between myocyte depolarisation and contraction. A number of studies have suggested that alteration of Ca^2+^ homeostasis is one of the possible mechanisms of cardiotoxicity [20,21]. The cardiotoxicity of doxorubicin is also associated with disturbance in cellular calcium homeostasis [22].

Here, we evaluated the potential cardiotoxicity of liensinine and neferine in neonatal rat CMs and hiPS-CMs. The resulting rapid cardiac dysfunction indicated that these two compounds exert significant inhibition of cardiac contraction and the CMs beating function recovered overtime and the recovery is time- and dose-dependent. Furthermore, inhibition effect to intracellular calcium induced by liensinine and neferine was observed and resulted in direct impairment of regular contraction on cardiomyocyte, which was associated with alterations in intracellular calcium handling.

## 2. Results

### 2.1. Optimization of Cardiomyocytes Seeding Density on the RTCA System

**Figure 1:**
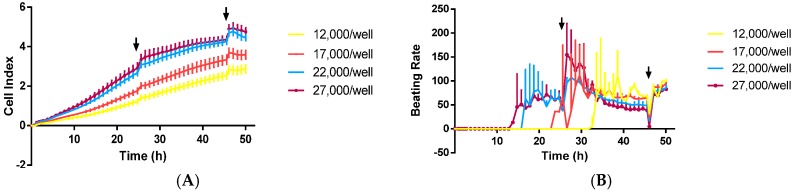

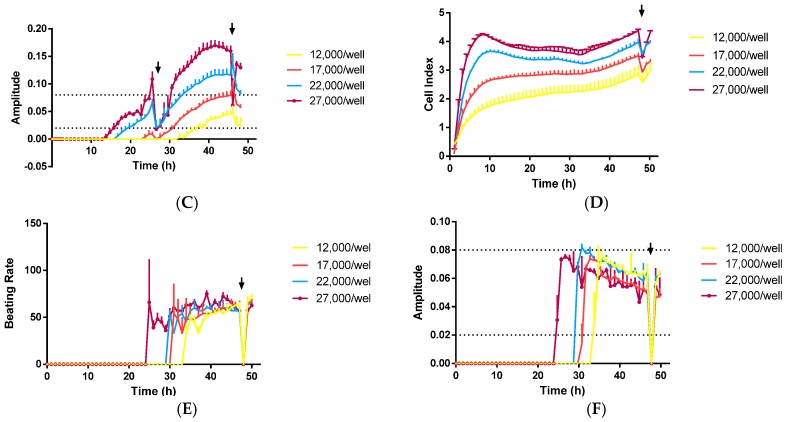
Optimization of Rat neonatal and hiPS cardiomyocytes seeding density on RTCA system. (**A**) Cell index; (**B**) Beating rate and (**C**) Amplitude of rat neonatal CMs within 48 h after seeding; (**D**) Cell index; (**E**) Beating rate and (**F**) Amplitude of hiPS-CMs. The black arrows indicated the time point of fresh medium addition. Data were presented as mean ± SD, *n* = 10.

Since the proper seeding density is the most important factor affecting contractility signals of the cardiomyocyte-based biosensor RTCA [23], we firstly optimized the cell seeding density. Rat neonatal CMs and hiPS-CMs were seeded at a density range from 12,000 to 27,000 cells/well on E-Plate 96. Basic CMs status was monitored for about 48 h to evaluate the performance of RTCA. No contractility signal was detected at 12 h, while regular beating patterns appeared within 48 h. CMs seeded at high density promote the adhesion to a greater extent than the low seeding density and seeding number was a positive influence on amplitude but negative influence on beating rate (Figure 1). Thus, the optimal seeding density in this study was determined to be 17,000 cells/well.

Once the contraction parameters of CMs become, the baseline beating rate prior to compound treatment was determined to be 113 ± 5 beats/min (mean ± SD, *n* = 16), 0.057 ± 0.006 (amplitude), for primary cultured rat CMs, and 63 ± 9 beats/min (mean ± SD, *n* = 16; beating rate), 0.048 ± 0.010 (amplitude) for hiPS-CMs.

### 2.2. Cardiotoxicity Assay Validation by Reference Compounds

**Figure 2:**
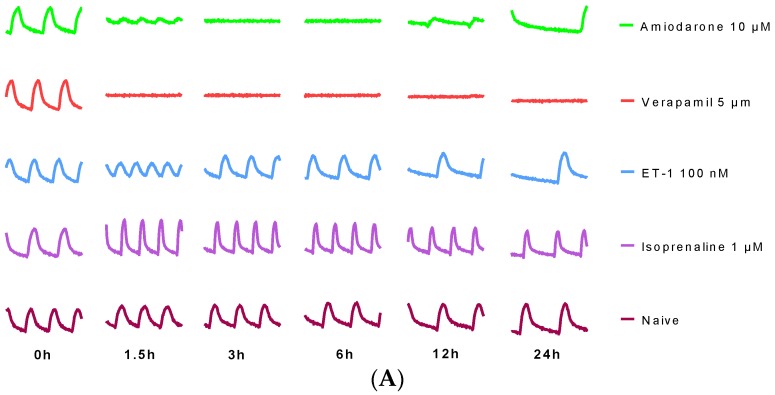

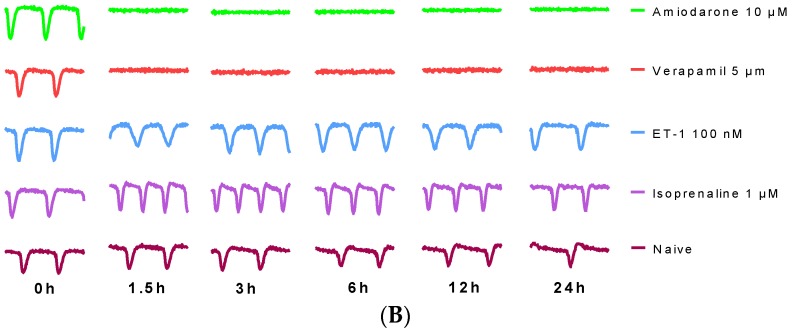
Representative contraction profiles of (**A**) rat neonatal CMs and (**B**) hiPS-CMs treated by reference compounds. Data were presented as mean ± SD, *n* = 3.

In order to evaluate the capability of the RTCA system to detect frequency and beating pattern changes, four reference compounds were employed to validate the assay. Positive inotropic compounds isoprenaline, endothelin-1 (ET-1), and negative inotropic compounds verapamil and amiodarone were tested on neonatal rat CMs and hiPS-CMs (Figure 2). Neonatal rat CMs and hiPS-CMs reached to the relevant concentrations with expected alterations in beating frequency: increased beat rate with the isoproterenol [24], ET-1 [25], and decreased beat rate with the amiodarone [26] and verapamil [27], which were consistent with reports from other groups. 

### 2.3. Contractile Effect of Liensinine and Neferine on Neonatal Rat CMs

**Figure 3:**
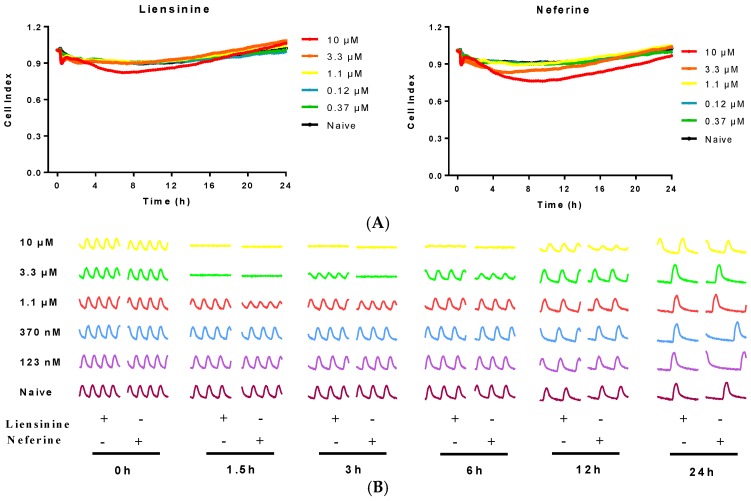

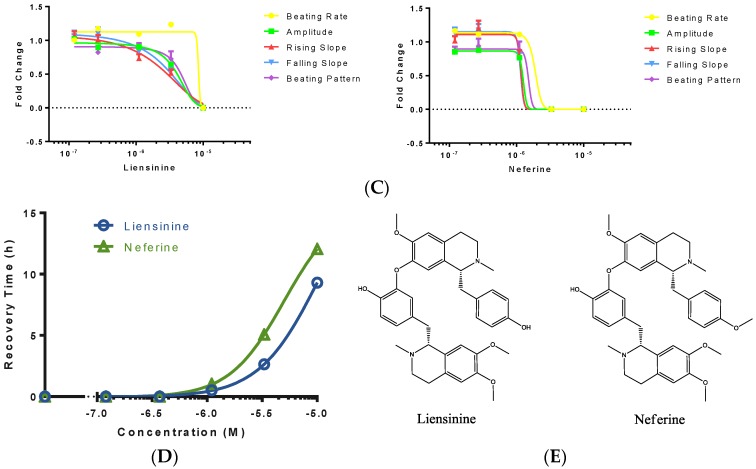
Typical rat neonatal CMs contraction profiles and dose responses to liensinine and neferine treatment. (**A**) Cell index fluctuation after various doses of liensinine treatment; (**B**) Temporal profiling of CMs beating status treated by various doses liensinine or neferine; (**C**) Dose response to liensinine and neferine of neonatal CMs 3 h after compounds treatment. Five CM beating parameters, including beating rate, amplitude, raising slop, falling slop and beating pattern, were used for evaluation; (**D**) Recovery time of beating rate of rat cardiomyocytes treated with various concentrations of tested compounds; (**E**) Chemical formula of liensinine and neferine. Data were presented as mean ± SD, *n* = 3.

The effect of liensinine and neferine was first evaluated on newly isolated neonatal rat CMs. Cells were treated with serial concentrations of liensinine and neferine post cell seeding 24 h. As shown in Figure 3A, Cell Index (CI) remained stable after addition of the two compound, which indicated that no cell death was induced by liensinine or neferine. Both compounds showed no cytotoxicity on neonatal rat CMs from the concentrations of 0.12 to 10 µM. At the same time, we monitored the effect of the two compounds on rat CMs contractility activities in real time. Before compound treatment, CM contractility was detected every minutes for at least 10 min to confirm the stability of the CMs. In Figure 3B, we show the CM contractile activities of 6 time-points before and after compounds treatment. Both liensinine and neferine had inhibitory effect on rat CM beating pattern in a concentration-dependent manner. Moreover, the CMs beating pattern recovered overtime and the recovery of CMs is time-dependent. Liensinine exerted significant inhibition on CMs contractility activity at 3.3 and 10 µM. Neferine showed a similar inhibitive manner on CMs except for longer inhibition (Figure 3B,D).

Furthermore, we analyzed the effect of liensinine and neferine on rat CMs with five detailed parameters, including beating rate, amplitude, rising slope, falling slope and beating period (Figure 3C). The beating rates of rat CMs are remain steady among most concentrations of liensinine treatment except for 10 µM, whose beating rate dropped to zero. The same sharp drop of beating rate was also observed after neferine treatment at high concentration. The amplitude, on the other hand, showed dose dependent decrease after liensinine and neferine treatment, with IC_50_ of 4.02 and 1.25 µM respectively. Rising slop and falling slop showed the same decrease pattern as amplitude. The beating pattern of CMs, which indicates the overall cardiomyocyte contractility, also showed dose dependent decrease after the two compounds treatment, with IC_50_ of 4.85 and 1.50 µM respectively. All these data indicated that liensinine and neferine mainly reduce amplitude other than cell beating rate in rat CMs. Moreover, both compounds affected the rat CMs’ amplitude without changing the shape of the contractility curve.

### 2.4. Contractile Effect of Liensinine and Neferine on hiPS-CMs

**Figure 4:**
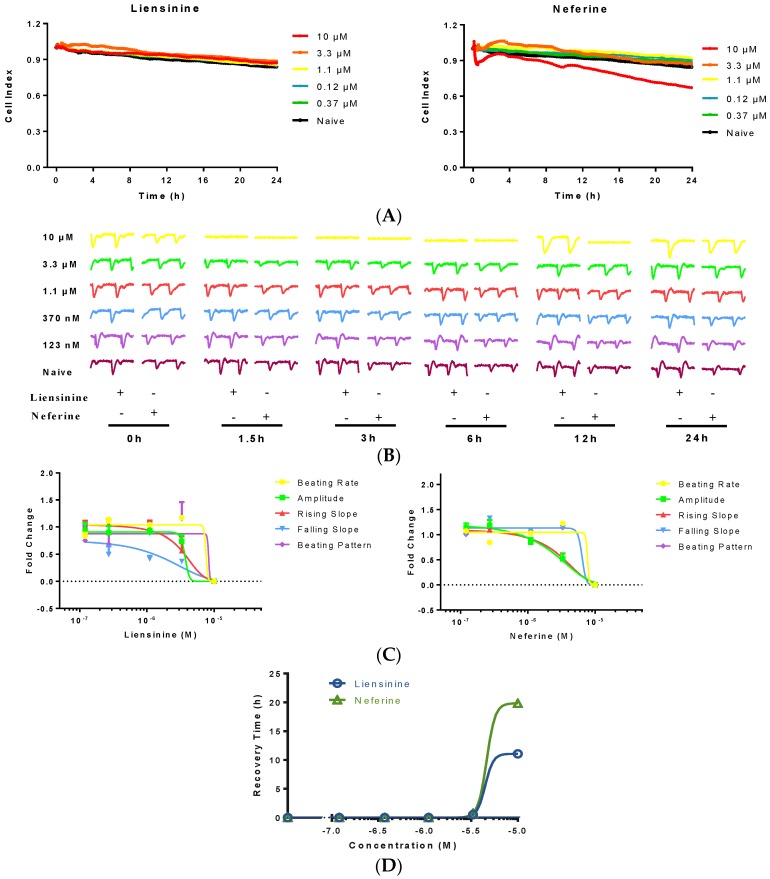
Typical contraction profiles and dose responses of rat neonatal and hiPS-CM to liensinine and neferine treatment. (**A**) Cell index fluctuation after various doses of liensinine treatment; (**B**) Temporal profiling of hiPS-CM beating functions treated by various doses of liensinine or neferine; (**C**) Dose response to liensinine and neferine of hiPS-CM 3 h after compounds treatment. Five hiPS-CM beating parameters, including beating rate, amplitude, raising slop, falling slop and beating pattern, were used for evaluation; (**D**) Recovery time of beating rate of hiPS-CM treated with various concentrations of tested compounds. Data were presented as mean ± SD, *n* = 3.

Considering the species difference and false negative and false positive on rat CMs [14], the cardiac toxicity of liensinine and neferine were further investigated on another model based on hiPS-CMs. The same concentrations of liensinine and neferine were applied on hiPS-CMs. As shown in Figure 4, the effect of liensinine and neferine on hiPS-CMs has some similarities compared to those in neonatal rat CMs, such as concentration dependent amplitude decrease and time dependent recovery. The CM contractile activities of 6 time-point before and after compound treatment are shown in Figure 4B. Both compounds showed no effect on beating rate, and mainly change CM amplitude at high concentration. A concentration dependent manner of inhibitory effect was also observed as expected on hiPS-CMs and the beating pattern recovered as time goes on. Moreover, the recovery time of neferine was generally longer than liensinine (Figure 4B,D), indicating that neferine possessed a stronger toxic effect than liensinine did. No cytotoxicity was observed after treatment of liensinine, while neferine showed slight cytotoxicity on hiPS-CMs at 10 µM (Figure 4A).

Meanwhile, different responses were also observed in hiPS-CMs. Neferine induced hiPS-CMs amplitude without changing CMs falling slop. The raising slop remained the same decreasing pattern as amplitude did. The IC_50_ (amplitude) of liensinine and neferine on hiPS-CMs is 3.69 and 1.29 µM respectively. All these data indicate that hiPS-CMs are more sensitive to liensinine and neferine than neonatal rat CMs are.

### 2.5. Effect of Liensinine and Neferine on Cell Cytotoxicity and Cell Viability

**Figure 5:**
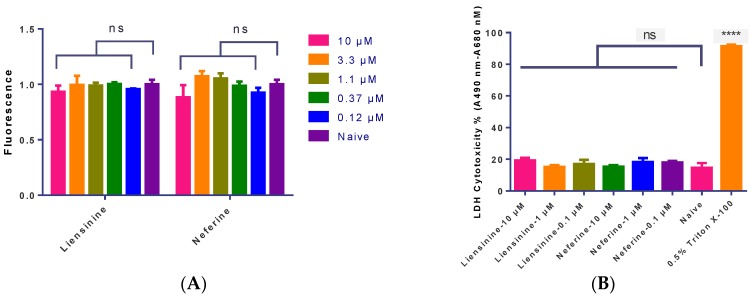
(**A**) A luciferase coupled ATP quantitation assay was used to determine the cytotoxicity of liensinine and neferine. Compounds were incubated in hiPS-CMs for 24 h before luminescence signal measurement; (**B**) Determination of LDH cytotoxicity of liensinine and neferine in hiPS-CMs. hiPS-CMs were plated in a 96-well plate in maintaining medium. Different concentrations of liensinine and neferine were added to the culture media and incubated for 24 h at 37 °C, 5% CO_2_. LDH cytotoxicity was measured using the Pierce LDH Cytotoxicity Assay Kit (Thermo Scientific, Hudson, NH, USA). Data were presented as mean ± SD, *n* = 3. ns, *p* > 0.05, **** *p* < 0.001.

CellTiter-Glo^®^ cell viability assay and released lactate dehydrogenase (LDH) cytotoxicity assay were applied to validate the data from RTCA label free assay. hiPS-CMs were incubated with different concentrations of liensinine or neferine (0.12 to 10 μM) for 24 h, and the cell viability was determined by luciferase coupled adenosine triphosphate (ATP) quantitation assay. As shown in Figure 5A, no significant cytotoxicity of liensinine and neferine was observed.

Meanwhile, released lactate dehydrogenase (LDH) cytotoxicity was further evaluated in this experiment. hiPS-CMs were treated with different concentrations of liensinine or neferine (0.1 to 10 μM) for 24 h, and LDH cytotoxicity was measured using the Pierce LDH Cytotoxicity Assay Kit (Thermo Scientific, Hudson, NH, USA). The results indicated that there are no significant differences on extracellular LDH between test and control groups within 24 h (Figure 5B). Both liensinine and neferine has no significant influence on cell viability using either ATP or extracellular LDH assays, which were consistent with the results obtained by RTCA assay.

### 2.6. Effect of Liensinine and Neferine on Intracellular Ca^2+^ in hiPS-CMs

Intracellular calcium ([Ca^2+^]_i_) is known to be a critical regulator of myocardial function, in which it plays a key role in maintaining cardiac excitation-contraction coupling. In order to investigate the effect of liensinine and neferine on CMs, intracellular Ca^2+^ changes were measured after compound treatment on hiPS-CMs.

**Figure 6:**
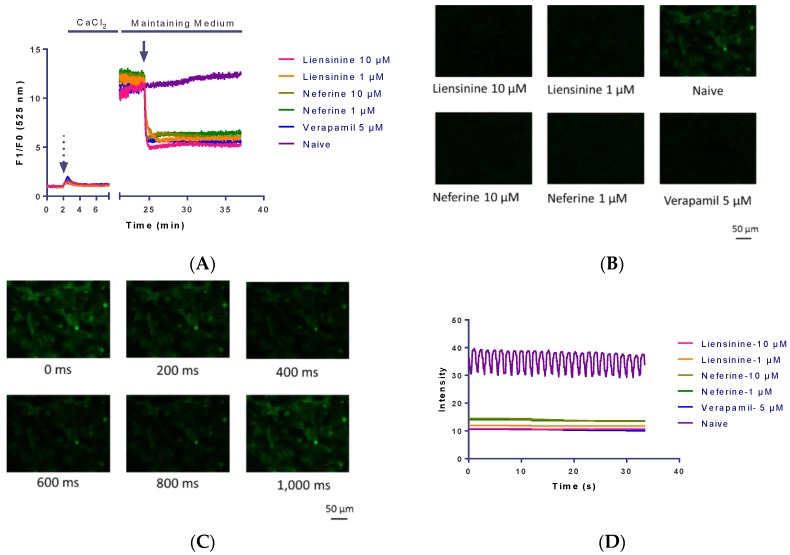
Effects of liensinine and neferine on [Ca^2+^]_i_ in hiPS-CMs. (**A**) Cardiomyocytes were loaded with Fluo 4 AM and pre-incubated in Ca^2+^ free HBSS for 30 min, then exposed to HBSS containing Ca^2+^ (1.2 mM final) The medium was exchanged with maintaining medium and incubated for 15 min. Similar to calcium channel blocker verapamil, liensinine and neferine decreased [Ca^2+^]_i_ in hiPS-CMs. Dashed arrow indicates CaCl_2_ addition and solid arrows indicate times of compound treatment. Horizontal axes in all traces show time in minutes. Measurements are indicated as a change in fluorescence after treatment divided by the initial fluorescence (F/F0); (**B**) Epifluorescence images show the [Ca^2+^]_i_ in 10 μM liensinine,1 μM liensinine, 10 μM neferine, 1 μM neferine and 5 μM verapamil addition; (**C**) A single calcium spark was performed within 1000 ms on hiPS-CMs. Images were shown consecutively 200 ms apart; (**D**) Temporal profiles of averaged calcium sparks at a typical single cardiomyocyte and the image were processed by Matrix Laboratory (MATLAB). Traces were scaled to the same peak fluorescence intensity.

The intracellular Ca^2+^ signal was determined in the presence and absence of extracellular Ca^2+^ to record the background fluorescence. The fluorescence intensity was recorded by FlexStation II. CaCl_2_ was added to the wells (contains 1.2 mM CaCl_2_) and fluorescence intensity recording continued. A transient increase in Ca^2+^ occurred. Treatment of cardiomyocytes with liensinine and neferine induced levels of [Ca^2+^]_i_ in the presence of maintaining medium. As shown in Figure 6A, the [Ca^2+^]_i_ decreased rapidly after compound treatment in hiPS-CMs, which was similar to calcium channel blocker verapamil (5 μM final concentration).

Epifluorescence images of calcium dye (Fluo 4 AM) was acquired by ImageXpress Micro XLS (Molecular Devices, Sunnyvale, CA, USA). The images showed the intensity of calcium decreased after liensinine (1 and 10 μM) or neferine (1 and 10 μM) treatment (Figure 6B). A typical calcium transient was observed when sparks rapidly increased in intensity and followed by contraction within 1000 ms (Figure 6C). Time-dependent fluorescence signal of single cell was further analyzed by Matlab, and the results of individual Ca^2+^ sparks demonstrated that liensinine and neferine inhibited the rhythmic calcium exchange (Figure 6D).

## 3. Discussion

Liensinine and neferine are two major isoquinoline alkaloids derived from embryo of *Nelumbo nucifera* Gaertner seeds, which account for over 0.2% of the seed. In T. Wei’s studies [28], they demonstrated that liensinine and neferine could antagonize the arrhythmias in rat. Embryo of *Nelumbo nucifera* Gaertner seeds has been widely used in Chinese food and tea. Using cardio RTCA system, we traced the dose and time responses of these two ingredients on contraction, beating rate, beating pattern, and recovery time in rat neonatal CMs and hiPS-CMs.

In primary neonatal rat CMs, we observed significant inhibitory response of liensinine on CM beating activities above 3 µM. Besides, cardiac dysfunction of rat neonatal CMs induced by liensinine could recover overtime. These data suggested that liensinine possessed a transient cardiotoxicity on rat CMs. The similar cardiotoxicity was observed after treatment of serial concentrations of neferine. However, the adverse potency of neferine was stronger and inhibitory effect lasted longer than that of liensinine. The IC_50_ of neferine was 1.25 µM on neonatal rat CMs. As shown in Figure 3E, neferine shares a similar chemical backbone with liensinine. The additional methyl group makes neferine more lipophilic. This property facilitates neferine much easier to be transported into cells and stay longer than liensinine did. Although time-dependent cardiotoxicity was observed in this study, the CI remain stable after compound addition under the concentration of 10 µM, which indicated that liensinine and neferine had no significant cytotoxicity on cardiomyocytes.

hiPS induced CMs have been widely used for evaluating the function of cardiomyocytes [29,30]. hiPS-CMs maintain a stable cardiac beating activity for over 2 months, which was supported for both acute and chronic studies of cardiac cell function [31]. Here, we employed hiPS-derived cardiomyocytes for further validating the function of liensinine and neferine. Unlike primary rat neonatal cardiomyocytes, the beating activity of hiPS-CMs was more stable, which was barely influenced by hiPS-CMs seeding-density or medium changing. The similar responses of CM beating activities were observed after treatment of the two compounds. However, hiPS-derived CMs are more sensitive to liensinine and neferine. The IC_50_ values of the two compounds are lower than those in primary rat CMs. Meanwhile, more time was required for hiPS-derived CMs to recover from cardiac dysfunction that induced by liensinine and neferine.

The impedance-based system (RTCA) has been proven to be a useful tool in a variety of assays, which including arrhythmia and compound validation [32,33], preclinical drug evaluation [34], monitoring compound effects [35], and cardiac disease models exploration [36,37]. Compared to cytotoxicity assays on H9C2 cell line [38], the RTCA cardio system provide a sensitive tool to detect potential cardiac side effects. Besides, the sensitive label-free assay made it possible to detect the regular beating pattern of cardiomyocytes under normal physiological conditions. Furthermore, the Cardio RTCA system provides multivariate statistics analysis, which uses regular beating patterns to reflect detailed beating status, to assess CM beating activities. In Malin K.B’s studies, the parameter beating rate and amplitude were used to detect frequency and beating pattern changes [39]. Beating rhythm irregularity was used to further quantify the effect of high dose of TER on iPS-CMs in Nguemo F’s research [40]. To analyze detailed beating status, multiple parameters need to be assessed. Thus, in this study, the shape of the beating pattern is constrained by five parameters, which including beating rate, amplitude, raising slope, falling slope and beating pattern.

In this study, the molecular mechanism of liensinine and neferine induced cardiotoxicity has been examined utilizing hiPS-CMs. The results revealed that liensinine and neferine resulted in a rapid and complete block of calcium transients, and the rhythmic calcium exchange was inhibited. In view of the importance of sarcoplasmic reticulum Ca^2+^-ATPase (SERCA2a) in the regulation of [Ca^2+^]_i_ during diastole, the expression of SERCA2a was also tested by immunostaining. No significant difference of SERCA2a was observed following different periods of compound treatment. It indicated that both liensinine and neferine can induce cardiotoxicity through disruption of calcium homeostasis. Specific targets will be tested in our further research.

## 4. Materials and Methods

### 4.1. Reagents and Materials

Dulbecco’s modified eagles medium (DMEM) and fetal bovine serum (FBS) for cell culture were purchased from Gibco BRL (Grand Island, NY, USA). Liensinine and neferine were purchased from National Institutes for Food and Drug Control (Beijing, China) with purities greater than 98%. Isoproterenol, amiodarone, verapamil and ET-1 were purchased from Sigma-Aldrich Chemicals (Sigma-Aldrich, St. Louis, MO, USA). All the chemicals were dissolved in dimethyl sulfoxide (DMSO) if not other stated.

### 4.2. Cell Culture of Cardiomyocytes

Primary neonatal rat cardiomyocytes were isolated from 24-h-old Sprague-Dawley rats of both sexes according to protocols published previously [13]. The cells were incubated in DMEM with 10% FBS at 37 °C, 5% CO_2_. Cell culture medium were refreshed every 2 days.

hiPS-CMs cells were obtained from CELLAPYBIO (Cat# CA2001106, Beijing, China), which were well validated cell line [41] and CMs were thawed from cryopreserved vials into CMs plating medium following recommended procedures. Briefly, each well of the E-Plate was pre-coated with 50 μL of a 1:100 diluted matrigel solution (BD) and maintained at 4 °C overnight. The cells were incubated in maintaining medium at 37 °C, 5% CO_2_. Cell culture medium were refreshed every 2 days.

### 4.3. CM Impedance Profiles

The xCELLigence RTCA cardio instrument was used to monitor the cardiomyocyte contractility. Impedance signals were recorded and displayed the data by converting into the cell index (CI) value, which correspond to a measure of relative changes in electric impedance and represents cell status. Hence, the attached cell number and their morphology were reflected by the CI value.

E-Plate 96 was coated with 50 μL of a 1:100 diluted matrigel solution (BD) and incubated overnight at 4 °C. Each well was replaced with 150 μL of CMs plating medium and then seeded at the density of 17,000 cell. The background impedance of media was determined before seeding the cells. The E-plate was monitoring every 15 min on the RTCA Cardio Instrument at 37 °C in a 5% CO_2_ incubator after incubated for 15 min at room temperature for an initial cells adhesion at the bottom of well. Typically, drug treatment was initiated 48–72 h after cell seeding depending on seeding density.

### 4.4. Compound Treatment on RTCA

The medium in Cardio E-Plate was replaced with DMEM or CMs Maintaining Medium 4 h before compound treatment. CMs were treated with various concentrations of compounds or vehicle control. Before compound treatment, the cells were sampled every minute for 15 min. After treatment, the sample frequency was every minute for the first 1 h and every 15 min for the next 23 h.

### 4.5. Cell Viability Assay

#### 4.5.1. ATP Depletion Assay

hiPS-CMs were seeded at 1.7 × 10^4^ per well into E-Plate and incubated in 5% CO_2_ at 37 °C. Different concentrations of the compound (10 to 0.12 μM) were added into E-Plate and incubated in 5% CO_2_ at 37 °C for 24 h. Luminescence was read by Envision 2100 multilabel reader to detect cells’ viability following incubation with CellTiter-Glo reagent (Promega, Madison, WI, USA) for 10 min. Each experiment was performed with three replicates.

#### 4.5.2. Lactate Dehydrogenase (LDH) Leakage Assay

hiPS-CMs were seeded at 1.7 × 10^4^ per well in 96-well plate. The cultured cells were treated with various concentration of compounds or vehicle and were incubated for another 24 h. the culture medium was aspirated and centrifuged at 1000× *g* for 10 min to obtain a cell free supernatant. LDH activity was examined using a commercially available kit (Thermo Fisher Scientific, Pittsburgh, PA, USA) following the manufacturer’s instructions. These measurements were performed with VersaMax (Molecular Device, Sunnyvale, CA, USA). The results were shown as fractions of LDH release and all the groups were compared to 0.5% Triton X-100. Data were presented as mean ± SD, *n* = 3.

### 4.6. Intracellular Calcium Transients on hiPS-CMs

CMs were plated 96-well plate on loaded with sensitive fluorescent dye Fluo-4 AM (4 μM), pluronic acid (0.02%) and probenecid (2 mM) in buffer for 30 min in the dark at room temperature. The background fluorescence was recorded for 1 min before adding CaCl_2_ (1.2 mM) to the wells and the signal was recorded for another 5 min at 37 °C on a FlexStation II (Molecular Devices). The medium was exchanged with maintaining medium and incubated for 15 min to stabilize the regular contraction of hiPS-CMs. The background fluorescence on maintaining medium was recorded for 5 min before compound/vehicle treatment. The change in fluorescence was calculated for each well using the respective F_0_ and F_1_ obtained for that well. The fluorescence intensity was calculated as F = F_1_/F_0_. Epifluorescence images were acquired on ImageXpress Micro XLS (Molecular Devices) and further analyzed with Matlab.

### 4.7. Data Analysis and Statistics

Data were analyzed using xCELLigence Cardio Software (Roche, Mannheim, Germany) and further analyzed with GraphPad Prism 6 (Graphpad Software, San Diego, CA, USA) or Matlab R2010b (Mathworks, Natick, MA, USA). Data were presented as mean ± SD. Statistical significance of differences was estimated by one-way ANOVA or Student’s *t* test. *p* < 0.05 (marked with an asterisk) was considered significant.

## 5. Conclusions

In conclusion, the current study clearly presents liensinine- and neferine-induced dose and time-related cardiotoxicity. We provide a paradigm to mimic cardiac regular contraction via *in vitro* phenotype to assess the potential cardiac risk of the tested compounds. Our findings produced novel insights into the cardiac safety thresholds of liensinine and neferine, which would call for more attention to embryos of Nelumbo nucifera Gaertner seeds and their application in Chinese medicine and tea. More importantly, this study introduced an efficient and effective approach to evaluate the cardiotoxicity of existing and novel drug candidates or food.

## Abbreviations

CMs: Cardiomyocytes; RTCA: real-time cell analysis system; hiPS-CMs: human induced pluripotent stem cell-derived cardiomyocytes; hiPS: human induced pluripotent stem; CI: Cell Index; LDH: lactate dehydrogenase; [Ca^2+^]_i_: intracellular calcium ion concentration; HBSS: Hanks’ Balanced Salt Solution; SERCA2a: sarcoplasmic reticulum Ca^2+^-ATPase.
